# Effects of space flight on sperm function and integrity: A systematic review

**DOI:** 10.3389/fphys.2022.904375

**Published:** 2022-08-11

**Authors:** Khulood Ahrari, Temidayo S. Omolaoye, Nandu Goswami, Hanan Alsuwaidi, Stefan S. du Plessis

**Affiliations:** ^1^ College of Medicine, Mohammed Bin Rashid University of Medicine and Health Sciences, Dubai, United Arab Emirates; ^2^ Gravitational Physiology and Medicine Research Unit, Division of Physiology, Otto Loewi Research Center of Vascular Biology, Inflammation, and Immunity, Medical University of Graz, Graz, Austria; ^3^ Division of Medical Physiology, Faculty of Medicine and Health Sciences, Stellenbosch University, Tygerberg, South Africa

**Keywords:** spaceflight, microgravity, sperm function, ionizing radiation, male fertility, countermeasures

## Abstract

With the advancement in space exploration and the intention to establish an inhabitable human settlement on Mars, it is important to investigate the effects of exposure to space/microgravity and the associated radiations on procreation. Sperm function and integrity are fundamental to male reproduction and can potentially be affected by the environmental changes experienced in space. Therefore, this study was conducted to systematically gather, filter, and collate all the relevant information on the effects of spaceflight on male reproductive parameters and functions. A search was performed utilizing the Preferred Reporting Items for Systematic Reviews and Meta-Analyses (PRISMA) guidelines. Data were extracted from the major electronic databases including PubMed, and other credible literature sources. MeSH search terms that were employed included “spermatozoa”, “microgravity”, and “ionizing radiation”. The literature search did not discriminate against papers published before a certain date due to the very limited number of articles available. However, there was a restriction on the male gender and language (English). The parameters included in this study are sperm motility, total sperm count, sperm DNA fragmentation hormonal levels and testicular histology. Following a comprehensive literature search, a total of 273 articles were retrieved and screened, 252 articles were excluded due to the irrelevance to the topic, duplication, and non-original articles. A total of 21 articles met the inclusion criteria and are included in the current study. Findings from these studies showed that sperm motility was decreased after exposure to microgravity and ionizing radiation. Total sperm count was also found to be reduced by microgravity only. Sperm DNA fragmentation was increased by both ionizing radiation and microgravity. Testosterone levels and testicular weight were also decreased by microgravity. Although there is a dearth in the literature regarding the effects of microgravity and ionizing radiation on male reproductive parameters, the available findings showed that exposure to microgravity poses a risk to male reproductive health. Therefore, it is essential to develop countermeasures to either manage, treat, or prevent these consequential adverse effects. Hence, this review also highlights some potential countermeasure approaches that may mitigate the harmful effects of microgravity and associated exposures on male reproductive health.

## Introduction

Space is the next habitat of interest that humans are exploring. With the recent agenda of different countries to colonize space, studies investigating the impact of microgravity/spaceflight and its associated exposures on the body system is increasing (Goswami et al., 2012; Goswami et al., 2013; Amjad et al., 2021; Goswami et al., 2021; Harris et al., 2022). Since procreation is an essential part of life sustainability, it is important to investigate the effects of microgravity on male reproduction, and specifically male fertility. One of the earliest studies reported that testes from rats flown on Cosmos 1887 presented with reduced testicular weight when compared to the vivarium controls (Sapp et al., 1990). The findings of Space-Lab three showed a reduction in the number of spermatogonial cells present in the seminiferous tubules of rat testes (Philpott et al., 1985). Following the discoveries of earlier studies, it became pertinent to investigate the exact impact of microgravity and the related exposures on male fertility, if humans are to sojourn in space. Utilizing spaceflight and simulated microgravity, studies have provided more insight regarding the impact thereof.

Hindlimb unloading or the tail suspension model is routinely used in animals to simulate the effects of gravity unloading on different physiological systems (Gao et al., 2018; Hawliczek et al., 2022). Using the tail suspension rat model, Hadley et al. reported that after 7 days of rat suspension without ligation of the inguinal canal, the architectural structure of the testis and epididymis were altered, as the seminiferous tubules became disorganized. Moreso, there was an accumulation of large multinucleated cells, and spermatids were seen in the lumen of epididymis (Hadley et al., 1992). The authors further reported an increase in the levels of serum luteinizing hormone (LH) and follicle-stimulating hormone (FSH), and a concomitant decline in serum testosterone and prolactin levels in the tail suspended rats. The presence of the accumulated immature spermatozoa in the epididymis suggests a possibility of the spermatids having not fully undergone the process of spermiogenesis, where there is an elongation of the nucleus and the condensation of chromatin. The latter is a consequence of the replacement of histones by arginine- and cysteine-rich protamine during spermiogenesis. Additionally, Zhang et al., using a rotating cell culture system, reported that spermatogonial stem cells (SSCs) co-cultured with Sertoli cells exhibited enhanced proliferation surpassing those cultured in static conditions, despite that the SSCs in simulated microgravity underwent an initial lag. After 14 days of culture, proliferating SSCs under simulated microgravity remained undifferentiated, although they maintained clone-forming capacity (Zhang et al., 2014). Meanwhile, another study reported that the number of duplicating cells in the tubules were significantly increased under simulated microgravity after testicular fragments isolated from prepubertal rats were cultured for 3 days in the rotating cell culture system (Ricci et al., 2004). Comparing the two findings, it is clear that simulated microgravity may enhance the proliferation of SSCs within the first few days of exposure but, the question remains if these cells would be able to undergo differentiation. Furthermore, Usik and Ogneva exposed mice to spaceflight for 21–24 days to measure the epigenetic changes. There were no differences observed in the level of cytoskeletal proteins, sperm-specific proteins and biomarkers for epigenetic changes. However, there were changes in the gene expression levels, as there was an increase in demethylase *Tet2* and a decrease in the histone deacetylase *Hdacl* (Usik and Ogneva, 2018)*.* This suggests that spaceflight may influence gene expression since the environment is known to affect genes. Other studies have also reported the impact of space flight/simulated microgravity on sperm functions and sperm parameters (Ogneva et al., 2020a; Ogneva, 2021). In addition to the effects of spaceflight and simulated microgravity on male fertility, studies have reported the impact of some of the exposures that come with microgravity, such as ionizing irradiation, on male fertility (Kamiguchi and Tateno, 2002; Barber et al., 2006; Santiso et al., 2012; Kodaira et al., 2017; Fuller et al., 2019; Wdowiak et al., 2019). Yan et al. reported that sperm DNA fragmentation and the expression of pro-apoptotic biomarkers were significantly higher in the groups exposed to microgravity and irradiation (Yan et al., 2013). Since microgravity and the accompanying exposures such as ionizing radiation may impact male fertility, this study aimed to analyse the literature systematically, bringing into light the evidence of the effect of spaceflight and ionizing radiation on sperm function and ultimately male fertility, putting both animal and human studies into consideration.

## Methods

A systematic review was conducted on the effect of space on sperm function and integrity in males using the Preferred Reporting Items for Systematic Reviews and Meta-Analyses (PRISMA) guidelines (PRISMA, 2020). The literature search did not discriminate against papers published before a certain date. This is due to the very limited number of articles available in the scientific literature on the effect of space flight on male reproductive parameters and sperm parameters. There was a restriction to the male gender, and articles written in the English language. Studies analyzed include human observational studies, animal models and *in vitro* experimental studies. Papers were excluded due to duplications, review articles, the lack of proper scientific basis or reliability of data collection.

**FIGURE 1:**
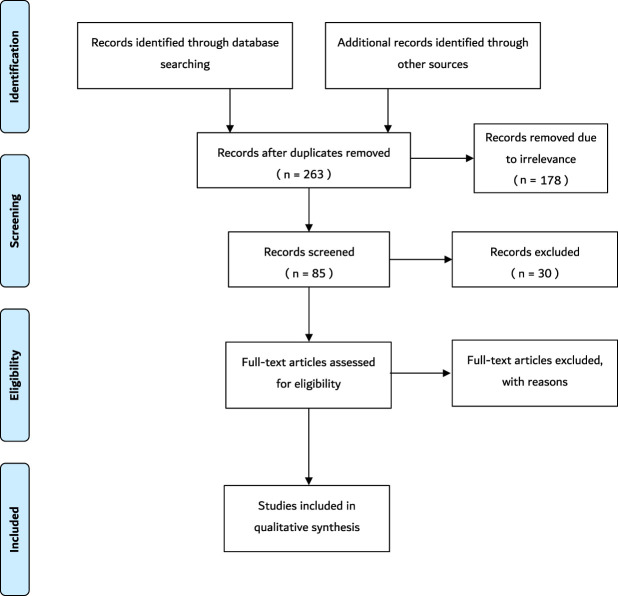
The schematic representation of the search method. Following the initial search, 273 records were retrieved. Ten duplicates were removed. Subsequently, 178 records were removed due to irrelevance after the titles were screened. Thereafter, 30 records were excluded due to the irrelevance of the abstracts. Finally, 34 full-text articles were removed because some did not satisfy the inclusion criteria and the others were not original articles. Thus, 21 articles that satisfied the inclusion criteria were included in the study. N = number.

Data extraction was conducted using the major electronic databases including PubMed, PubMed Central, MedLine, Google Scholar and Cochrane. In addition, other credible literature sources were also explored. Structured literature searches were performed based on specific keywords and inclusion criteria. MeSH search terms and keywords that were used include “sperm”, “space”, “space flight”, “sperm function”, “sperm motility”, “microgravity”, “space radiation”, “human body”, “spermatozoa”, “ionizing radiation”, and any synonyms of these words. Boolean operators–AND, OR, NOT–were also used. The title and abstract were screened at first to confirm that the topics match. Thereafter, the entire article was screened to further confirm the relevance of the article to the current study. Data extracted from each paper satisfying the eligibility criteria were used for this study. The variables include study author and publication year, study setting, sample characteristics, studies exploring the effect of cosmic/ionizing radiation on sperm parameters, studies exploring effects of anti/micro-gravity on sperm parameters, and any other study that showed the effects of environmental changes of space on sperm. The PRISMA flowchart summarizing the data collection process is presented in Figure 1.

## Results

During the initial search, 273 records were identified. Ten duplicates were removed. Subsequently, 178 records were removed due to irrelevance after the titles were screened. Thereafter, 30 records were excluded due to the irrelevance of the abstracts, such as studies that are not within the scope of the study. Finally, 34 full-text articles were removed because some did not satisfy the inclusion criteria and the others were not original articles. Thus, 21 articles that satisfied the inclusion criteria were added in the current study. A summary of the key information from these articles is shown in Table 1. These articles included five studies from Japan (23.8%), four studies from China (19.04%), four studies from Russia (19.04%), three studies from the United States (14.2%), one study from India (4.76%), one study from the United Kingdom (4.76%), one study from Australia (4.76%), one study from Egypt (4.76%), and one study from Spain (4.76%) (Tash and Bracho, 1999; Tash et al., 2002; Kamiya et al., 2003; Sasaki et al., 2004; Ikeuchi et al., 2005; Ding et al., 2011; Kumar et al., 2013; Li et al., 2013; Yan et al., 2013; Zhou et al., 2016; Wakayama et al., 2017; Olejnik et al., 2018; Usik and Ogneva, 2018; Fuller et al., 2019; Matsumura et al., 2019; Ogneva et al., 2020a; Boada et al., 2020; Ogneva et al., 2020b; Said et al., 2020; Ogneva, 2021). Seventeen of the articles conducted the experiment on Earth (fifteen simulations and two occupational exposures), three of the studies performed the experiment on the International Space Station (ISS), and one of the articles conducted experiments during a Space Shuttle mission. Seventeen of the articles included experiments performed on animals, which include mice (N = 224), rats (N = 48), echinogammarus marinus (N = 72), Gammarus Pulex (N = 72), rams (N = 12), and *Drosophila melanogaster* (N = 560). The remainder of the articles included experiments conducted on humans (N = 285). All articles mentioned the age group except for six articles (Sasaki et al., 2004; Xi et al., 2016; Fuller et al., 2019; Ogneva et al., 2020a; Ogneva et al., 2020b; Said et al., 2020). Four articles did not mention the sample size (Tash et al., 2002; Ogneva et al., 2020b; Said et al., 2020; Ogneva, 2021), but in the remaining studies, the reported sample sizes ranged between 12 and 560 subjects. Thirteen articles were exclusively performed on microgravity (Tash and Bracho, 1999; Tash et al., 2002; Kamiya et al., 2003; Sasaki et al., 2004; Ikeuchi et al., 2005; Ding et al., 2011; Masini et al., 2012; Usik and Ogneva, 2018; Matsumura et al., 2019; Ogneva et al., 2020a; Boada et al., 2020; Said et al., 2020; Ogneva, 2021), while seven articles were performed on ionizing radiation (Kumar et al., 2013; Li et al., 2013; Zhou et al., 2016; Wakayama et al., 2017; Olejnik et al., 2018; Fuller et al., 2019; Ogneva et al., 2020b), and only one article conducted combined experiments on both microgravity and ionizing radiation (Yan et al., 2013).

**TABLE 1:** Overview of findings included in the current study. Studies are grouped into animal and human section. N/A = not available, M = male, LH = luteinizing hormone, FSH = follicle-stimulating hormone, g= gram, kg = kilogram.


Author (year)	Country	Sample size	Subjects	Sex	Age	Average weight	Study setting	*In Vivo/In Vitro*	Model	Exposure	Results						
											Sperm motility	Total sperm count	Hormones			Sperm DNA fragmentation	Testicular weight and architecture/histology
													**Testosterone**	**LH**	**FSH**		
**Animals**																	
Ding et al. (2011)	China	48	Rats	M	8 weeks	200–250 g	Simulation on Earth	*In Vivo*	Tail suspension	Simulated Microgravity	N/A	Decreased	N/A	N/A	N/A	N/A	N/A/Disorganized
Fuller et al. (2019)	United Kingdom	72	Echinogammarus Marinus	M	Unknown	Unknown	Simulation on Earth	*In Vivo*	Beta emitter phosphorus-32	Ionizing Radiation	N/A	No change	N/A	N/A	N/A	Increased	N/A
		72	Gammarus Pulex	M							N/A	No change	N/A	N/A	N/A	N/A	N/A
Kamiya et al. (2003)	Japan	34	Mice	M	≥10 weeks	25.6 g	Simulation on Earth	*In Vivo*	Tail suspension	Simulated Microgravity	Decreased	N/A	Decreased	N/A	N/A	N/A	Decreased/Disorganized
Li et al. (2013)	China	36	Mice	M	12 weeks	30–35 g	Simulation on Earth	*In Vivo*	Carbon ion beam irradiation	Ionizing Radiation	N/A	N/A	N/A	N/A	N/A	N/A	Disorganized
Masini et al. (2012)	United States	10	Mice	M	8 weeks	Unknown	ISS	*In Vivo*	Space	Microgravity	N/A	N/A	N/A	N/A	N/A	N/A	Disorganized
Matsumura et al. (2019)	Japan	12	Mice	M	5 weeks	Unknown	ISS	*In Vivo*	Space	Microgravity	Decreased	No change	N/A	N/A	N/A	N/A	No change
Olejnik et al. (2018)	Australia	12	Ram Lambs	M	14 weeks	22.6 kg	Simulation on Earth	*In Vivo*	Linear accelerator producing photon beam	Ionizing Radiation	N/A	Decreased	N/A	N/A	N/A	N/A	No change/Disorganized
					20 weeks	27.5 kg					N/A	Decreased	N/A	N/A	N/A	N/A	No change/Disorganized
Ogneva et al. (2020)	Russia	Unknown	Mice	M	Unknown	Unknown	Simulation on Earth	*In Vitro*	Random positioning machine	Simulated Microgravity	Decreased	N/A	N/A	N/A	N/A	N/A	N/A
Ogneva et al. (2020)	Russia	560	Fruit Fly	M	2 days	Unknown	Simulation on Earth	*In Vivo*	Random positioning machine	Simulated Microgravity	Increased	N/A	N/A	N/A	N/A	N/A	N/A
Ogneva et al. (2021)	Russia	Unknown	Fruit Fly	M	2 days	Unknown	Simulation on Earth	*In Vitro*	Random positioning machine	Simulated Microgravity	Increased	N/A	N/A	N/A	N/A	N/A	N/A
		21	Mice	M	2 weeks						Decreased	N/A	N/A	N/A	N/A	N/A	N/A
Said et al. (2020)	Egypt	Unknown	Rats	M	Unknown	120–150 g	Simulation on Earth	*In Vivo*	Gamma Cell-40 irradiator (Cesium-137)	Ionizing Radiation	Decreased	N/A	Decreased	N/A	N/A	N/A	Decreased/Disorganized
Sasaki et al. (2004)	Japan	15	Mice	M	Unknown	25.8 g	Simulation on Earth	*In Vivo*	Tail suspension	Simulated Microgravity	N/A	N/A	Decreased	N/A	N/A	N/A	No change
Tash et al. (1999)	United States	Unknown	Sea Urchin	M	Unknown	Unknown	Space Shuttle Missions	*In Vitro*		Simulated Microgravity	No change	N/A	N/A	N/A	N/A	N/A	N/A
Tash et al. (2002)	United States	Unknown	Rats	M	13–15 months	Unknown	Simulation on Earth	*In Vivo*	Tail suspension	Simulated Microgravity	N/A	Decreased	No change	No change	No change	N/A	Decreased/Disorganized
Usik et al. (2018)	Russia	42	Mice	M	Unknown	28 g	Simulation on Earth	*In Vivo*	Tail Suspension	Simulated Microgravity	No change	Decreased	N/A	N/A	N/A	N/A	Decreased
Wakayama et al. (2017)	Japan	12	Mice	M	3 months	Unknown	ISS	*In Vitro*	Freeze-dried spermatozoa	Ionizing Radiation	N/A	N/A	N/A	N/A	N/A	No change	N/A
Yan et al. (2013)	China	42	Mice	M	10 weeks	30–35 g	Simulation on Earth	*In Vivo*	Tail suspension and Carbon Ion beam irradiation	Simulated Microgravity and Ionizing Radiation	N/A	Decreased	N/A	N/A	N/A	Increased	N/A
**Humans**																	
Boada et al. (2020)	Spain	15	Humans	M	26–40 years	Unknown	Simulation on Earth	*In Vitro*	Parabolic flight	Simulated Microgravity	No change	No change	N/A	N/A	N/A	No change	N/A
Ikeuchi et al. (2005)	Japan	18	Humans	M	22–40 years	Unknown	Simulation on Earth	*In Vitro*	Parabolic flight	Simulated Microgravity	Decreased	N/A	N/A	N/A	N/A	N/A	N/A
Kumar et al. (2013)	India	134	Humans	M	21–50 years	Unknown	Earth	*In Vivo*	Occupationally exposed	Ionizing Radiation	Decreased	No change	N/A	N/A	N/A	Increased	N/A
Zhou et al. (2016)	China	118	Humans	M	28.26 ± 3.12 years	78.13 kg	Earth	*In Vivo*	Occupationally exposed	Ionizing Radiation	Decreased	No change	N/A	N/A	N/A	Increased	N/A

Note: N/A = not available, M = males, ISS, international space station.

## Discussion

### Evidence of the effect of spaceflight/microgravity and ionizing radiation on sperm function: Animal studies

#### Sperm motility

Kamiya et al. performed a study on 34 mice using the tail suspension method to simulate the microgravity environment of space. The tail suspension method is a test in which the hind limbs of mice/rats are suspended by taping their tails to a raised bar, in such a position that they cannot escape or hold on to nearby surfaces (Can et al., 2011), thus creating a simulated microgravity environment. After 7 days of exposure to microgravity, there was a significant decrease in the average path velocity (31.2 ± 10.7 μm/s versus 42.0 ± 6.5 μm/s), mean track speed (54.7 ± 18.8 μm/s versus 68.1 ± 10 μm/s), and mean progressive velocity (18.3 ± 8.8 μm/s versus 24.5 ± 5.4 μm/s), when compared to the control. These are sperm kinematic parameters, which are used to evaluate the percentage of sperm motility (Kamiya et al., 2003). Matsumara et al. caged 12 mice in the ISS for 35 days, thereafter, spermatozoa were retrieved for *in vitro* fertilization (IVF), to evaluate the effects of microgravity on fertilization. Some parameters of sperm motility were reduced, but that did not affect the ability of the sperm to fertilize the oocyte (Matsumura et al., 2019). In another study, the sperm samples of the experimental mice were isolated from the epididymis and were subjected to simulated microgravity using a random position machine for 1 h, 3 h, and 6 h. The random positioning machine provides 3D multidirectional rotation relative to gravity vector, so that the superposition of the orientation vectors of the objects in the gravitational field is equal to zero per minute, on average (van Loon, 2007). Under the microgravity exposure, the speed of movement did not change after 1 h and 3 h, but it significantly decreased by 32% after 6 h of exposure *p* < 0.05 (Ogneva et al., 2020a). From the study of Ogneva et al., the sperm motility of 2-day old fruit flies and 21 male mice (12 weeks) was evaluated after exposure to a random positioning machine for 6 h. The results obtained indicated that stimulated microgravity leads to a decrease in the speed of movement of mouse spermatozoa by 29% (*p* < 0.05). In contrast, the speed of sperm of the fruit fly after exposure to simulated gravity increased significantly by 30% (Ogneva, 2021). This is also supported by another study by the same author, which was carried out on 560, 2-day old, fruit flies, exposed to a random positioning machine. The results also showed an increase in the speed of movement of the sperm tails by 34% (58.5 ± 2.6 μm/s, *p* < 0.05) (Ogneva et al., 2020b). We can see that the motility of the spermatozoa of insects and mammals under microgravity conditions changes in different ways. It is assumed that the effect of simulated microgravity on the motility of mammalian spermatozoa is mediated through the regulation of phosphorylation and that of insects through the regulation of dephosphorylation of motor proteins that form the axoneme of the sperm tail (Tash and Bracho, 1999; Ogneva, 2021). Nevertheless, Tash et al. who obtained the sperm of male sea urchins and sent them on a Space Shuttle mission for microgravity exposure (Tash and Bracho, 1999), as well as Usik et al. who subjected 42 mice to 30 days of tail suspension to simulate weightlessness (Usik and Ogneva, 2018), reported that there were no significant changes in the sperm motility. The reason for this controversy is not well understood, as there is still a lot of uncertainty and much to be explored.

Regarding irradiation and its effect on sperm motility, Said et al. investigated the underlying mechanism of alpha-lipoic acid (LA) against testicular damage caused by irradiation. The whole body of male rats were exposed to radiation and then administered LA. When rats were exposed to radiation, the sperm motility results showed a significant decrease compared to that of the controls (Said et al., 2020).

#### Sperm count

Yan et al. used the tail suspension method and carbon ion beam irradiation to simulate a microgravity environment and space ionizing radiation, respectively and investigated its effect on the testis. The carbon ion was equipped with a passive beam delivery system (Li et al., 2007). Compared with the control group, a disordered arrangement of spermatogenic cells was observed and the number of spermatids was significantly smaller in the group exposed to both microgravity and ionizing radiation (*p* < 0.05) (Yan et al., 2013). In the study of Olejnik et al., three irradiation doses (9, 12, 15 Gy) were administered to 24 ram lambs, aged 14 weeks (Group 1, n = 12) and 20 weeks (Group 2, n = 12), by linear accelerator producing photon beam, then testicular biopsies were collected 1,2, and 3 months after irradiation. At 1 month after irradiation, the total sperm count of both groups were significantly decreased *p* < 0.05. Two months post irradiation, the total sperm count of both groups were similar to controls. After 3 months of radiation, Group 1 had less spermatogonia per cross section than controls (∼3% versus 10%, *p* < 0.05). Group 2, the higher radiation treatment group (12Gy) had lower spermatogonia per cross section compared to the controls (9.6% versus 12.7%, *p* < 0.05) (Olejnik et al., 2018). The initial large reduction in the numbers of spermatogonia were not permanent as recovery began at 2 months after irradiation. The pattern of change in spermatogonia number over time differed between the two groups. Spermatogonia number in Group 2 decreased rapidly and then increased steadily over the next. 2 months to become similar to the controls, but spermatogonia numbers from Group 1 increased at 2 months after irradiation and then decreased again and was significantly lower than that of controls at 3 months. This pattern of increase and then decrease is also reported by Kangasniemi et al. (Kangasniemi et al., 1996). The reason behind this may be due to the fact that radiation may have induced a defect in spermatogonial stem cells that limits self-renewal to repopulate depleted seminiferous tubules. In the study of Usik et al., 42 mice were subjected to 30 days of tail suspension to simulate weightlessness. Results obtained showed that number of sperm decreased significantly by 45% (*p* < 0.05) (Usik and Ogneva, 2018). This may be due to damage to the testes architecture which will further be discussed in the “testicular architecture/histology” section. In contrast, a study that was performed on the ISS evaluated the total sperm count. Results showed no significant differences, as it was comparable to that of the control group (Matsumura et al., 2019). This finding is consistent with Fuller et al., where two types of male crustaceans, Echinogammarus marines and Gammarus pulex, were exposed to ionizing radiation by beta emitter phosphorus-32 at dose rates of 0, 0.1, 1, and 10 mGy/d. Sperm quality parameters were assessed using a fluorescent staining method. In E. marines as well as Gammarus pulex, no significant effect of radiation dose rate on sperm numbers was recorded (Fuller et al., 2019).

#### Sperm DNA fragmentation

The study of Yan et al. used tail suspension and carbon ion beam irradiation to simulate microgravity and ionizing radiation respectively on 42 mice. It was reported that the SDF index was significantly increased in the animals from the experimental group compared to those in the control group (21.35 ± 0.78% versus 9.54 ± 0.31%, *p* < 0.001) (Yan et al., 2013). The SDF index refers to the percentage of sperm with DNA strand breaks in comparison to the total number of sperm analysed (Xi et al., 2016). In addition, Fuller et al., who obtained two types of male crustaceans and exposed them to ionizing radiation by beta emitter phosphorus-32 recorded the occurrence of DNA damage. DNA damage was assessed using single cell gel electrophoresis. Radiation dose rate had a significant *p* < 0.05 effect on DNA damage in E. marines spermatozoa. DNA damage increased as dose rate increased (10 mGy/d treatment 20.69 ± 12.74% versus 10.30 ± 7.51%). No DNA damage assessment was done on Gammarus pulex (Fuller et al., 2019). However, in Wakayama et al., freeze-dried mouse spermatozoa of 12 mice were held on the ISS for 9 months at –95°C and then examined after exposure to space radiation. During the study period, irradiation from space to the sample case was ∼100 times higher than that on Earth (Yatagai et al., 2011). The DNA damage in the space sperm samples was slightly increased compared with the control samples kept on Earth, irrespective of mouse strain, but this was regarded as insignificant since the slight damage in the sperm DNA during space preservation was repaired by the oocyte cytoplasm during IVF and did not impair the birth rate or normality of the offspring (Wakayama et al., 2017).

#### Hormone levels

Kamiya et al. also examined the changes in testosterone levels by using the tail suspension method to evaluate the possibility of spermatogenesis failure in a microgravity environment (Kamiya et al., 2003). The tail suspension model is considered to be a model of body fluid shift (Royland et al., 1994), and a few studies have used tail suspended rats as a spermatogenesis failure model. To date, these studies have demonstrated decreases in the serum testosterone level (Hargens et al., 1984; Hadley et al., 1992). The testosterone level was significantly lower in the tail-suspended group (0.74 ± 1.28 ng/ml, *p* < 0.05) compared with controls (2.38 ± 3.50 ng/ml) in this study. The decrease in testosterone corresponded with the finding of Merrill et al., who examined changes in the serum electrolyte and hormone levels in spaceflight and tail-suspended rats (Merrill et al., 1992). The decrease in testosterone suggests that testosterone secretion is reduced because of a reduction in the testicular blood flow associated with the cranial shift of body fluids (Murashev et al., 1988). In addition to the previous study, Sasaki et al. that used tail suspension methods as well to simulate microgravity on 15 mice noticed that testosterone levels were also decreased (tail suspended group: 0.67 ± 1.21 ng/ml, control group: 3.35 ± 5.09 ng/ml, *p* < 0.05) (Sasaki et al., 2004). Said et al. investigated the underlying mechanism of alpha-lipoic acid (LA) against testicular damage caused by irradiation. Exposure to radiation triggered a significant decrease in both estradiol and testosterone levels by 48% and 64% respectively compared to the control group. Treatment with LA significantly increased estradiol levels; however, it had no effect on testosterone levels (Ogneva et al., 2020b). Nonetheless, in the study from Tash et al., circulating testosterone, FSH, and LH levels were normal at 6 weeks in the tail suspended animals and not significantly different from free-roaming control animals. The results were as follows: testosterone (tail suspended rats at 1,347 ± 156 pg/ml versus control at 1,099 ± 5 80 pg/ml, *p* < 0.001), FSH (tail suspended rats at 119 ± 15 ng/ml versus controls at 162 ± 38 ng/ml), and LH (tail suspended rats at 7.6 ± 1.0 ng/ml versus controls at 6.9 ± 1.6 ng/ml). In this study, tail suspended rats were partly ligated at the inguinal canal to prevent the testes from descending into the abdomen and thus diminishing the effect of the tail suspension (Tash et al., 2002).

#### Testicular weight and architecture

Ding et al. used the tail-suspension model to simulate microgravity and investigated the effect of microgravity on the tissue structure and function of the testis in sexually mature male rats. Tail suspension was achieved with a tail harness suspending the limbs above the floor of the cage according to the method of Wronski and Morey-Holton (Wronski and Morey-Holton, 1987). Forty-eight male Wistar rats weighing 200–250 g were randomly assigned to three groups (N = 16 each): control, tail traction, and tail suspension. In the tail traction group, the rats were places in a tail-lift harness without suspension of the hindlimbs above the floor. After the rats were suspended for seven or 14 days, the testes were evaluated by histological and electron microscopic methods. Results of the control and the tail suspended rats were included. Upon histopathological observation of the testis by light microscopy, there were almost no observation of spermin the seminiferous tubules. Degeneration and necrosis of the spermatogenic cells were seen, seminiferous tubules were arranged sparsely, surface membrane appeared rough and disordered, interstitial tissue showed edematous, fibrotic, and haemorrhagic changes. Tail suspension caused severe damage to the seminiferous tubules making the basal lamina appear rough. Ding et al. also mentioned that with extended suspension time, the damage to the testes became more serious even reaching irreversibility (Ding et al., 2011). Tash et al. who used the tail suspension method to produce a microgravity environment showed a significant reduction in the number of testicular sperm and elongating spermatids accompanied the decline in testicular weight in the tail suspended animals (1.10 ± 0.11 g, *p* < 0.001) versus the control group (1.70 ± 0.06 g). After 6 weeks of tail suspension, spermatogenesis was significantly reduced to the extent that no spermatogenic cells beyond round spermatids were present in the testis and no normal mature spermatozoa were found in the epididymis (control: 7.82 ± 0.46 sperm x 106/ml, tail suspended: 1.04 ± 0.24 sperm x 106/ml) (Tash et al., 2002). Kamiya et al. simulated the microgravity environment of space and observed that after 7 days of tail suspension, testicular weight was significantly different between the tail suspended mice (93.22 ± 11.31 mg, *p* < 0.05) and control mice (98.53 ± 12.17 mg); however, body weight did not change (Kamiya et al., 2003). This study also looked at the histological architecture of the testes in depth using the light microscopy with hematoxylin and eosin (H&E) and periodic acid–Schiff (PAS) staining. The microscopy showed impairment of spermatocytes beyond the pachytene stage (third stage of prophase meiosis). Almost no spermatozoa were found in the lumen. Multi-nucleated giant cells were occasionally seen. The histology showed hypo-spermatogenesis, as well as loss of all spermatogenic cells, including spermatogonia. The histologic appearance of the Sertoli cells and interstitial Leydig cells appeared to be unaffected in hind limb suspended animals. Another study reported that after Wistar rats were flown for 22 days onboard the biosatellite Cosmos-605, there were no morphological changes in the testes after 24–48 h and 26–27 days postflight, and that the offspring of male rats that were exposed to 22-days weightlessness did not differ from the controls with respect to the number of the new-born, birthweight, weight gain during the first postnatal month, and resistance to hypoxia (Plakhuta Plakutina et al., 1976). However, Macho et al. reported an increase in plasma corticosterone and insulin levels in male rats after space flights for a period of 7, 15, 18 and 20 days. Plasma levels of growth hormone were decreased and those of epinephrine and norepinephrine were elevated in rats exposed to longer space flights (18 or 20 days). This suggests that exposure of rats to space flight is followed by changes in plasma metabolic hormonal levels, but the sympathetic-adrenomedullary system is only slightly activated by longer space flights (Macho et al., 1993). Li et al. investigated the mechanism of action of heavy ion radiation on mouse testes. The testes of 36 male mice aged 12 weeks subjected to whole body irradiation with carbon ion beam (0.5 and 4 Gy) were analyzed at 7 days after irradiation. The histological changes showed cavity formation, disarranged spermatogenic cells, and disrupted basement membrane, disordered and shrunk seminiferous tubules, and thinning seminiferous epithelium (Li et al., 2013). Masini et al. housed 10 mice in the Mouse Drawer System (MDS) developed by the Thales-Alenia Space Italy (Cancedda et al., 2002). The MDS, loaded with the mice, was launched in the Space Shuttle Discovery within the Space Transport System (STS)-128 mission, on 28 August 2009, for exposure to microgravity. It was then housed in the Japanese Experimental Module (Kibou) on the ISS until its return to the Earth by Space Shuttle Atlantis (STS-129 mission) on 27 November 2009. Only three mice returned to the Earth alive after 91 days of space flight. Testes and the epididymis were sampled bilaterally from each mouse killed by inhalation of carbon dioxide at the Life Sciences Support Facility of Kennedy Space Center within 3–4 h after landing and were either processed or frozen immediately. The sections were stained by the H&E staining method and investigated by using an inverted light microscope. The histology showed degenerative changes. This included disorganization and a slight reduction in the thickness of the spermatogenic cells. In addition, sloughing of cells in the lumen, separation of germ cells from the basal laminae and vacuolation of the germinal epithelium were observed. The interstitial tissue displayed inflammatory exudates. More seminiferous tubules were shrunken and distorted (Masini et al., 2012). From the study of Olejnik et al., three irradiation doses (9, 12, 15 Gy) were administered to 24 ram lambs aged 14 weeks (Group 1, n = 12) and 20 weeks (Group 2, n = 12) by linear accelerator producing photon beam, then testicular biopsies were collected at 1,2, and 3 months after irradiation. During the third month, testicular weights (42 ± 6 g) were similar to that of controls (65 ± 13g, *p* = 0.137). Three months post irradiation, in Group 1, there were less spermatogonia per tubule cross section (3 spermatogonia per cross section at 15Gy, 2.9 at 12Gy, 3.2 at 9 Gy compared to 10 at controls, *p* < 0.05). In Group 2, the 12Gy treatment had less spermatogonia per tubule cross section (9.6 versus 12.7, *p* < 0.05) (Olejnik et al., 2018). Said et al. reported testicular damage caused by Gamma Cell-40 irradiator emitting Cesium-137. Testicular weight was reduced in rats exposed to ionizing radiation compared to controls (1.74 ± 0.16 g versus 2.47 ± 0.27g, *p* < 0.05). Testicular histology showed significant morphological alteration in germinal epithelium of the seminiferous tubules associated with atrophy, reduction in the size of the seminiferous tubules, and vacuolation with few spermatozoa seen in the lumen. Moreover, the germinal epithelium has been detached from the basement membrane. Vascular congestion and increased extracellular matrix in the interstitial spaces accompanied with edematous regions, and haemorrhage were prominent in the irradiated group (Ogneva et al., 2020b). These findings were consistent with previous studies (Li et al., 2013; Usik and Ogneva, 2018). Usik et al.. Who subjected 42 mice to 30 days of tail suspension to simulate weightlessness, observed a decrease in testicular weight of the experimental group (177 ± 16mg, *p* < 0.05) compared to the control group (259 ± 20 mg) (Usik and Ogneva, 2018). Nevertheless, the results of another study showed that there was no difference in the weight of the testes following exposure to microgravity (4.01 ± 0.29 g) compared to the control group (3.82 ± 0.25 g) (Matsumura et al., 2019). These findings were consistent with Sasaki et al. (tail suspended group: 94.2 ± 11.2 mg, control group: 98.5 ± 12.2 mg) (Sasaki et al., 2004).

### Evidence of the effect of spaceflight/microgravity and ionizing radiation on sperm function: Human studies

#### Sperm motility

Parabolic flight is a flight that produces an almost gravity-free state for 20–25 s aboard a jet airplane in parabolic flight at a height of 29,000–21,000 feet (Osborne et al., 2014). Ikeuchi et al. collected semen samples from 18 men and exposed the spermatozoa to parabolic flight. Parabolic flight is a flight that produces an almost gravity-free state for 20–25 s aboard a jet airplane in parabolic flight at a height of 29,000–21,000 feet (Ikeuchi et al., 2005). Following exposure, the percentage of total sperm motility (26.85 ± 21.60% versus 45.33 ± 23.98%, *p* = 0.004), and mean progressive motility (18.53 ± 17.34% versus 32.15 ± 23.35%, *p* = 0.007) were significantly decreased. Studies of Kumar et al. and Zhou et al., included men who were chronically exposed to ionizing radiation due to their occupation, this includes healthcare workers (Kumar et al., 2013; Zhou et al., 2016). Kumar et al. included 134 male volunteers of which 83 were occupationally exposed to ionizing radiation and 51 were non-exposed control subjects. The motility characteristics were markedly lower between the experimental and the control groups (*p* < 0.001) (Kumar et al., 2013). Zhou et al. included 118 subjects of which 46 men were occupationally exposed to ionizing radiation and 72 men were not (the control group). The occupationally exposed men were from various hospitals with diagnostic radiation facilities (mainly computed tomography) (Zhou et al., 2016). All 46 men had operated the equipment for more than 2 years and were considered to have been chronically exposed to low-dose radiation, as reported by Kumar et al. Sperm motility was significantly lower in the exposed men compared to the non-exposed men (20.85 ± 3.41% vs 25.19 ± 3.60%, *p* < 0.001), sperm morphology was abnormal as well (10.04 ± 3.36% vs 16.71 ± 5.67%, *p* < 0.001) (Zhou et al., 2016). Boada et al. collected sperm samples from 15 normozoospermic healthy donor. These samples were preserved in cryostraws and stored in a secure and specific nitrogen vapor cryoshipper. They were then subjected to parabolic flights with an aerobatic single-engine aircraft capable of providing parabolas of up to 8–9 s of microgravity, which is significantly different from other parabolic flight studies. This methodology was previously described by Perez-Poch et al. (Perez-Poch et al., 2016). The sperm motility of frozen samples exposed to microgravity and control samples showed comparable results. There were no statistically significant differences in the percentage of sperm motility (frozen samples at 21.83 ± 11.69 versus controls at 22.54 ± 12.83) (Boada et al., 2020). Other studies that exposed ejaculated sperm to parabolic flights reported a decrease in total sperm motility (control 45.33 ± 23.98 versus microgravity 26.85 ± 21.60% (*p* = 0.004), and progressive motility (control 32.15 ± 23.35 versus microgravity 18.53 ± 17.34% (*p* = 0.007)), and thought that the reason behind this might be due to chemical changes in the intracellular environment during microgravity exposure (Ikeuchi et al., 2005). However, Boada et al. suggested that the undetectable differences observed in the parameters analyzed could be that the effects of microgravity on sperm motility was minimized because the samples were frozen and sperm integrity was shielded by cryoprotectants.

#### Sperm count

Boada et al. reported that the exposure of healthy donors to parabolic flight did not affect sperm count, as no significant changes between the experimental and control groups (39.01 ± 32.02 × 10^6^/ml versus 39.29 ± 36.53 × 10^6^/ml) was observed. This result reinforces the previous hypothesis that sperm is protected against microgravity by freezing them in comparison to the fresh ones (Boada et al., 2020). Additionally, Kumar et al. showed that after exposure to occupational ionizing radiation, the total sperm count was not affected, as there was no statistically significant difference between the exposed and non-exposed men (64.16 ± 4.40 × 10^6^/ml versus 68.44 ± 5.98 × 10^6^/ml) (Kumar et al., 2013). This was supported by Zhou et al. who stated that there were no statistically significant differences in sperm concentration between the exposed and the non-exposed men (Zhou et al., 2016).

#### Sperm DNA fragmentation

Both Kumar et al. and Zhou et al. studied men who were chronically exposed to ionizing radiation due to their occupation and both studies had the same outcome. Kumar et al. reported that the level of SDF was significantly higher in the exposed group as compared to the non-exposed group (*p* < 0.05–0.0001). These findings showed that exposure to occupational radiation may have a profound implication on the fertility and reproductive outcome of health workers (Kumar et al., 2013; Zhou et al., 2016). Zhou et al. reported that the SDF index of exposed subjects was 29.43 ± 4.57% and non-exposed subjects 14.68 ± 6.32% (*p* < 0.001). These findings show that exposure to occupational radiation may have a profound implication on the fertility and reproductive outcome of health workers, and importantly, on the health of the children born to such fathers since the spermatozoa carrying nuclear abnormalities can fertilize the oocytes (Marchetti et al., 1999), and the embryos thus derived from the irradiated sperm carry substantial risk of trans-generational genomic instability (Shimura et al., 2002; Adiga et al., 2010). In space, there is exposure to ionizing radiation and therefore the exposure could also potentially carry risk of trans-generational genomic instability. On the contrary, Boada et al. showed that there were no significant changes. Samples exposed to microgravity had a mean of 13.33 ± 5.12% sperm fragmentation, while control samples had a mean of 13.88 ± 6.14%. The mean percentage of SDF was similar in both groups (Boada et al., 2020).

## Summary

Having critically and systemically analyzed the literature, and deducting evidence from both animal and human studies, it is safe to say exposure to microgravity and associated ionizing irradiation causes hypogonadism, hyposteriodogenesis, decreased sperm function and sometimes transgenerational gene mutation in animals. However, in humans, although studies have shown hyposteriodogenesis as a consequence of exposure to microgravity, report about its effect on sperm quality and quantity are contradictory. Some studies reported decrease in sperm quality such as motility while some showed no changes. This trend is also seen in the reports of sperm quantity such as sperm count and semen volume. These contradicting outcomes on sperm functional parameters may be because of different mode of exposure, method of semen collection (*in vivo* (samples collected from men placed under microgravity) versus *in vitro* (samples collected from healthy donors)), the duration, and the age differences in the different set of cohorts. Similarly, aspects related to social interactions and psychology, which play important roles in reproduction physiology, could also have contributed to the contradictory results. Future studies related to male reproduction should carefully integrate these confounding variables.

Despite these conflicting outcomes on sperm parameters in humans, sex hormones are often reduced, and this can hamper spermatogenesis. To give a perspective on how spermatogenesis can be adversely impacted; under microgravity, the body orientation is not usually straight and the blood flow to the testes maybe further reduced, causing decreased oxygenation. Under a normal erect body position, blood flow to the testis is lesser when compared to other organs (Rawy et al., 2021). The testes receives its blood supply through the testicular artery, having high flow resistance and hence resulting in a lower intra-testicular capillary pressure and slightly higher venous pressure than other organs (Sweeney et al., 1991; Rawy et al., 2021).

Most evidence suggests that the testis is particularly susceptible to vascular system disruption and that testicular dysfunction can result from moderate blood supply disruption (Damber and Bergh, 1992; Bergh and Damber, 1993). As in every other organ, blood supply must be strictly mediated, this is particularly significant for the testis since the oxygen concentration in the seminiferous tubules is very low (Setchell, 1990). Therefore, any decrease in blood supply causes ischemic harm that may lead to sperm deterioration. Partial restriction of the testicular artery has been reported to have an adverse effect on the development, volume and histological structure of bull testes, resulting in complete or incomplete arrest of spermatogenesis (Kay et al., 1992). Several studies in humans (Battaglia et al., 2001; Biagiotti et al., 2002; Tarhan et al., 2003) and rats (Bergh et al., 2001) have shown an association between testicular blood flow and quality of sperm. Moreover, hormones appear to be involved in the mediation of testicular blood flow (Macho et al., 1993; Ogneva et al., 2016; Rawy et al., 2021; Abdelnaby, 2022). This is evidenced by the report of Rawy et al., that following administration of GnRH to rams, the arterial pulsatile index (PI) and resistance index (RI) decreased for about 120 h, and serum testosterone concentration was negatively correlated with both PI and RI (Rawy et al., 2021). This observation was also reported in bulls, with the inclusion that nitric oxide was also increased (Abdelnaby, 2022). Hence, reduced hormonal levels seen upon exposure to microgravity and associated irradiation could cause testicular dysfunction by altering testicular blood flow and disrupting the normal functioning of the HPG axis.

Evidence that exposure to microgravity poses a risk to male reproductive health has been provided thus far. Since it is essential to maintain and preserve fertility for successful space exploration and colonization, countermeasures must be set in place to either manage, treat or prevent any adverse effects that can be imposed due to exposure to microgravity or associated irradiation.

## Potential counter measures to the effect of microgravity on sperm function

Researchers have been able to identify some of the adverse effects of microgravity on various physiological systems, and this includes sperm function. In counteracting these negative consequences, studies have focused more on the use of pharmacological methods, exercise, improving dietary supplementation and creating artificial gravity. Though these measures are directed towards improving physiological functions, the question abates whether these strategies can also improve male reproductive health?

Therefore, this section will briefly discuss some of the impending approaches that may be employed to prevent, treat, or alleviate the negative consequences of microgravity on male reproductive health. This includes cryopreservation of sperm before take-off, gene silencing, telomere length preservation, exercise, improved dietary supplementation, inducing artificial gravity, and the provision of devices that may protect against ionizing irradiation.

### Cryopreservation

Sperm cryopreservation is extensively used in infertility treatment and fertility preservation in cancer patients, which has consequently resulted in millions of live births from these patients (Yu et al., 2021). Sperm cryopreservation can be performed by either slow freezing or vitrification. Slow freezing has been successfully employed and widely used at fertility clinics performing the different assisted reproductive techniques (ART) (Jang et al., 2017). Vitrification which is a process of solidifying liquid into an amorphous or glassy state has become a faster alternative method of sperm cryopreservation with significant benefits regarding simple equipment and applicability to fertility centers (Tao et al., 2020).

Boada et al. showed in their study that frozen sperm samples preserved in cryostraws and stored in a nitrogen vapour cryoshipper do not suffer significant alterations after microgravity exposure. The results of motility, morphologically normal spermatozoa and the SDF index were comparable between the control and microgravity groups. The comparability may be due to the placement of the cells. The frozen cells were in a near-dormant state, and this may cause reduced cellular metabolism due to the reduced temperature. Therefore, the lack of differences seen between frozen samples exposed to microgravity and those maintained in-ground state affords the possibility of considering the safe transport of human male gametes to space (Boada et al., 2020). Since both gametes and embryos can be cryopreserved, then, in the advent of sojourning in the space, this method can be utilized.

### Gene silencing

The application of gene silencing has received great attention in recent years. This phenomenon, ‘gene silencing’, is utilized as a defense mechanism against invasive nucleic acids. For the knockdown of gene expression, the process of RNA interference (RNAi) must be initiated. RNAi is a biological process by which double-stranded RNA (dsRNA) induces sequence-specific gene silencing by targeting mRNA for degradation (Han, 2018). When RNA are double-stranded, the body regards them as foreign, and are cut into smaller segments (small interfering RNA (siRNA)) by the nuclease Dicer, which then triggers RNAi response to induce gene silencing or degradation. In research, gene-specific, synthetic siRNA can be used to induce gene silencing. In 2001, Elbashir et al. showed that transfection of synthetic 21 base-pair siRNA duplexes into mammalian cells efficiently silences endogenous gene expression in a sequence-specific manner (Elbashir et al., 2001). This finding heralded the use of siRNA for gene silencing in mammalian systems.

This technology can be integrated into the study of microgravity. This can be carried out by initiating RNAi response in genes that their expression under microgravity/spaceflight or any associated irradiation could cause detrimental effects on male reproductive health. For instance, in the studies of Usik et al. and Ogneva et al. where some genes were mal-expressed upon exposure to microgravity (Usik and Ogneva, 2018; Ogneva et al., 2020a), the process of gene silencing may prove to be a useful tool to alleviate the adverse effects thereof.

### Telomere preservation

Telomeres, situated at the end of a chromosome are made of repetitive sequences of non-coding DNA that protect the chromosome from damage. Luxton et al. reported that telomere lengths were increased during spaceflight irrespective of mission duration and that its length rapidly shortened upon return to Earth (Luxton et al., 2020a). Upon completion of the experiment, the telomeres’ length of astronauts that were exposed to microgravity was shorter than it previously was. They also showed that telomere length was positively correlated to oxidative stress (OS) and increase frequencies of chromosomal inversions. The same authors reported, in a different study, that in addition to increased OS and inflammation, there was persistent DNA damage observed with telomeric and chromosomal aberrations after exposure to microgravity (Luxton et al., 2020b).

Although studies have not yet identified any association between telomere length and altered male reproduction function. However, the occurrence of OS and inflammation reported under microgravity and accompanying radiation, which are also known mediators in the pathogenesis of male infertility, makes the strategy of preserving telomere length viable. The indirect preservation can be performed by preventing OS through implementing antioxidant therapy in astronauts during spaceflight, as this may counteract the negative effect of OS on telomere length.

### Exercise and improved diet

It is widely known that exposure to microgravity induces changes in the physiological functions of the body. In counteracting these adverse effects, it was suggested that exercise and diet supplement implementation may be critical roles (Hides et al., 2019). For instance, Hides et al. suggested that since exercise improves the symptoms of patients with lumbopelvic and spinal muscle pain (low back pain), then exercise should be encouraged in astronauts, since exposure to microgravity causes low back pain and some other skeletal defects.

Furthermore, since sufficient energy intake is required to maintain anabolic processes necessary for normal bone growth support and remodelling, it is suggested that diets that can improve calcium bioavailability be supplemented. Calcium bioavailability can be enhanced by reducing dietary alkalinization, restricting dietary sodium and preventing dietary calcium oxalate urolithiasis. Fettman described in detail the importance of incorporating dietary management when getting exposed to microgravity (Fettman, 2001). These processes of improved lifestyle have been shown to have positive impact on male reproductive function.

## Conclusion and recommendation

The current study systematically reviewed the effects of microgravity and ionizing radiation on sperm motility, total sperm count, sperm DNA fragmentation, hormone levels, and testicular architecture/histology, and how it affects male fertility. Although some studies reported the negative effect of microgravity on sperm motility, some showed no effect, while others indicated that normal sperm motility was restored post-exposure. It remains evident that exposure to microgravity and ionizing radiation can adversely affect spermatogenesis and as well alter sperm DNA/chromatin integrity. Since the mature human spermatozoa do not have the capability to repair their DNA, sperm DNA damage depending on the radiation dose may lead to permanent infertility, and/or increase the risk of congenital anomalies occurrence in the offspring.

Recommendations for future research would be to perform experiments and observe the effects of space flight on the male reproductive system during longer space missions utilizing both *in vitro* and *in vivo* studies. Researchers should also begin to perform more experiments on human subjects as part of their studies since these findings will provide a true reflection of the results. With plans for significantly longer space missions and ultimately colonization, it is vital to consider multi-generational survival under microgravity. Furthermore, studies that will focus on developing and implementing countermeasure strategies should be designed, as this will help in reducing the futuristic consequences of exposure to microgravity and associated irradiation.
